# Association of PIN3 16-bp duplication polymorphism of *TP53* with breast cancer risk in Mali and a meta-analysis

**DOI:** 10.1186/s12881-020-01072-4

**Published:** 2020-07-03

**Authors:** Brehima Diakite, Yaya Kassogue, Guimogo Dolo, Oumar Kassogue, Mamadou Lassine Keita, Brian Joyce, Erin Neuschler, Jun Wang, Jonah Musa, Cheick Bougari Traore, Bakarou Kamate, Etienne Dembele, Sellama Nadifi, Mercy Isichei, Jane L. Holl, Robert Murphy, Seydou Doumbia, Lifang Hou, Mamoudou Maiga

**Affiliations:** 1 Faculty of Medicine and Odontostomatology, University of Technical and Technological Sciences of Bamako (USTTB), 1805, Point G, Bamako, Mali; 2 University Teaching Hospital Point G, Bamako, Mali; 3 Preventive Medicine Department, Cancer Epidemiology and Prevention, Northwestern University, Chicago, IL 60611 USA; 4 Institute for Global Health, Northwestern University, Chicago, IL 60611 USA; 5 Department of Radiology, College of Medicine, University of Illinois at Chicago, Chicago, IL 60612 USA; 6 Department of Obstetrics and Gynecology, Faculty of Medical Sciences, University of Jos, Jos, Plateau State Nigeria; 7 Hassan II Univesity Aïn chock, Casablanca, Morocco; 8 Department of Neurology, The University of Chicago, Chicago, IL 60637 USA

**Keywords:** Breast cancer, *TP53*, PIN316-bp duplication, Meta-analysis, Malian population

## Abstract

**Background:**

Breast cancer, the most common tumor in women in Mali and worldwide has been linked to several risk factors, including genetic factors, such as the PIN3 16-bp duplication polymorphism of *TP53*. The aim of our study was to evaluate the role of the PIN3 16-bp duplication polymorphism in the susceptibility to breast cancer in the Malian population and to perform a meta-analysis to better understand the correlation with data from other populations.

**Methods:**

We analyzed the PIN3 16-bp duplication polymorphism in blood samples of 60 Malian women with breast cancer and 60 healthy Malian women using PCR. In addition, we performed a meta-analysis of case-control study data from international databases, including Pubmed, Harvard University Library, Genetics Medical Literature Database, Genesis Library and Web of Science. Overall, odds ratio (OR) with 95% CI from fixed and random effects models were determined. Inconsistency was used to assess heterogeneity between studies and publication bias was estimated using the funnel plot.

**Results:**

In the studied Malian patients, a significant association of PIN3 16-bp duplication polymorphism with breast cancer risk was observed in dominant (A1A2 + A2A2 vs. A1A1: OR = 2.26, CI 95% = 1.08–4.73; *P* = 0.02) and additive (A2 vs. A1: OR = 1.87, CI 95% = 1.05–3.33; *P* = 0.03) models, but not in the recessive model (*P* = 0.38). In the meta-analysis, nineteen (19) articles were included with a total of 6018 disease cases and 4456 controls. Except for the dominant model (*P* = 0.15), an increased risk of breast cancer was detected with the recessive (OR = 1.46, 95% CI = 1.15–1.85; *P* = 0.002) and additive (OR = 1.11, 95% CI = 1.02–1.19; *P* = 0.01) models.

**Conclusion:**

The case-control study showed that PIN3 16-bp duplication polymorphism of *TP53* is a significant risk factor for breast cancer in Malian women. These findings are supported by data from the meta-analysis carried out on different ethnic groups around the world.

## Background

Breast cancer as a multifactorial disease is the most diagnosed cancer among women worldwide [1]. The incidence of breast cancer in women would be higher in developed countries due to the great heterogeneity in terms of polymorphism frequency, proportion of deletions and insertions, but with the recent improvements and availability of diagnostic infrastructure in LMICs, the detection rate has continued to increase. Over the past decade, the number of women globally affected has increased, but data from LMICs are still limited [2]. With the advent of genomics, dramatic advances have been made in breast cancer research. Recent report showed that in addition to clinical, lifestyle and environmental risk factors, an individual’s genetic background plays a crucial role in the development of breast cancer [3]. Several genes have been shown to be associated with an increased risk of breast cancer, such as damaged DNA repair genes (*BRCA1 and BRCA2), tumor protein p53* (*TP53*), *Checkpoint kinase 2 (CHEK2), methylenetetrahydrofolate reductase* (*MTHFR*)*, fibroblast growth factor receptor 2 (FGFR2) and glutathione S-transferase mu 1* (*GSTM1*) [4]. *TP53*, a tumor suppressor gene, is involved not only in the development of breast cancer, but also in the development of other human cancers. Indeed, this gene plays a significant role in the response to stress. The protein TP53, also called the genome guardian, is a transcription factor that controls the expression of many genes involved in cell cycle regulation, DNA repair, cell death and senescence [5–8]. The great heterogeneity reported in the *TP53* in breast cancer may be linked to the geographic origin and ethnic differences of patients [8–10].

The *TP53* is located on the chromosome 17p13.1 [11] and consists of 12 exons (https://www.ncbi.nlm.nih.gov/gene/7157). It is highly polymorphic both in exonic and intronic regions with more than 200 polymorphisms (http://www-p53.iarc.fr/). Of these, p.Arg72Pro, p.Pro47Ser and PIN3 16-bp duplication of *TP53* are the most studied polymorphisms because of their critical roles in modifying the function and/or expression of TP53 [7, 12]. Sequence changes in the coding region affected by 16 bp duplication of PIN3 may result in impaired function and expression of p53 [13]. This disturbance is involved in the etiopathology of many cancers, including breast cancer [14, 15]. Several studies around the world have found an association between the polymorphisms of this gene and the development of breast cancer [16, 17], while others have found no effect [18–20]. It has been reported in developed countries that individuals harboring the A2A2 genotype or 16-bp duplication in intron 3 of *TP53* are at increased risk of breast cancer [21, 22]. However, very few studies have been performed in Africa populations [19], especially in Mali. The literature review revealed that the association between the PIN3 16 bp duplication polymorphism and the risk of breast cancer has not been evaluated in our population. Consequently, we carried out the present work in order to understand firstly the relation between the duplication PIN3 16 bp and the development of breast cancer in the Malian population and secondly to carry out a comparative meta-analysis of different studies around the world to better estimate the risk of breast cancer.

The literature review showed that the relationship between PIN3 16-bp duplication polymorphism and the risk of breast cancer has not been evaluated in our population. Therefore, we carried out the present work in order to understand firstly the relation between PIN3 16-bp duplication and the risk of breast cancer in the Malian population and secondly to perform a comparative meta-analysis of different studies around the world better to estimate the risk of breast cancer.

## Methods

### Case control study

#### Subject selection and sample collection

The study was approved by the ethics committee of the Faculty of Medicine and Odontostomatology (2018/63/CE/FMPOS) at the University of Sciences, Techniques and Technologies of Bamako (USTTB). The study was explained to each participant prior being asked to sign the approved Informed Consent.

Sixty women (mean age 43.72 ± 3.14) with clinically and histologically diagnosed breast cancer and 60 age-matched apparently healthy women (mean age 43.90 ± 2.92) from the general population were recruited at the University Hospital Center (CHU) of Point G in Bamako, Mali, between July 2018 and July 2019. All cases had early stage cancer (stage II). Clinico-pathological parameters including age at diagnosis, localization, use of contraceptive, menopausal status, parity, breastfeeding, family history of breast cancer, history of benign breast disease, obesity, smoking, histological type, tumor size, nodal involvement and metastasis were collected from each patient’s medical record. In the control group, the inclusion criteria were all Malian women aged of 18 years or over coming from the general population of whom no chronic disease has ever been diagnosed (such as cancer, diabetes, etc.) and having accepted informed consent. Healthy subjects with a history of breast cancer, chronic diseases such as diabetes, or other types of cancer were excluded as controls. A total Five milliliter of peripheral blood was collected from each participant in an EDTA tube for thegenotyping analysis of PIN3 16-bp duplication polymorphism of *TP53*.

#### Genotyping of PIN3 16-bp duplication

Qiagen’s GentaPuregene Extraction Kit was used to extract the genomic DNA from white blood cells. DNA quantity and quality were determined by spectrophotometer. Genotyping of PIN316-bp duplication polymorphism was performed by allele specific PCR (AS-PCR) using published primers previously described [17, 19, 23, 24]. A final reaction volume of 25 μl containing 12.8 μl buffer, 1.5 μl MgCl2, 1.5 μl dNTPs, 1.0 μl primers, 2.0 μl Taq DNA polymerase, and 2.0 μl genomic DNA was used to amplify the PIN3 16-bp duplication of the *TP53*. PCR amplification conditions were previously described by Maarouf and al [19].. The PCR products after electrophoresis on a 4.5% agarose gel showed a fragment of 119 bp for the A1 allele (wild type or no duplication) and a fragment of 135 bp for the A2 allele (Insert or 16-bp duplication).

#### Statistical analysis

SPSS 11.0 was used to analyze the data. Chi-square tests (two-sided) were performed to evaluate the correlation between the PIN3 16-bp duplication and the clinical and histological features. Hardy-Weinberg equilibrium for the PIN3 16-bp duplication genotype distribution of *TP53* was tested by Chi2 analysis with exact probability. An odds ratio (OR) test with 95% confidence interval (CI) and P <0.05 was used to determine the association between PIN3 16-bp duplication polymorphism of *TP53* and the risk of breast cancer, according to the different genetic models (dominant: A1A2 + A2A2 vs. A1A1, recessive: A2A2 vs. A1A2 + A1A1 and additive: A2 vs. A1). The *P* value < 0.05 was considered significant.

### Meta-analysis study

#### Literature search

The keywords “*TP53*”, “Intron 3 Ins16 bp or PIN3 16-bp duplication”; “Polymorphism or mutation or genes” and “breast cancer” were used to perform a literature search of Pubmed, Harvard University Library, Genetics Medical Literature Database, Genesis Library and Web of Science. Only articles published in English were retained. Additional articles were identified by examining the references cited in articles and reviews retained from the search.

#### Article inclusion c***riteria***

**Fig. 1:**
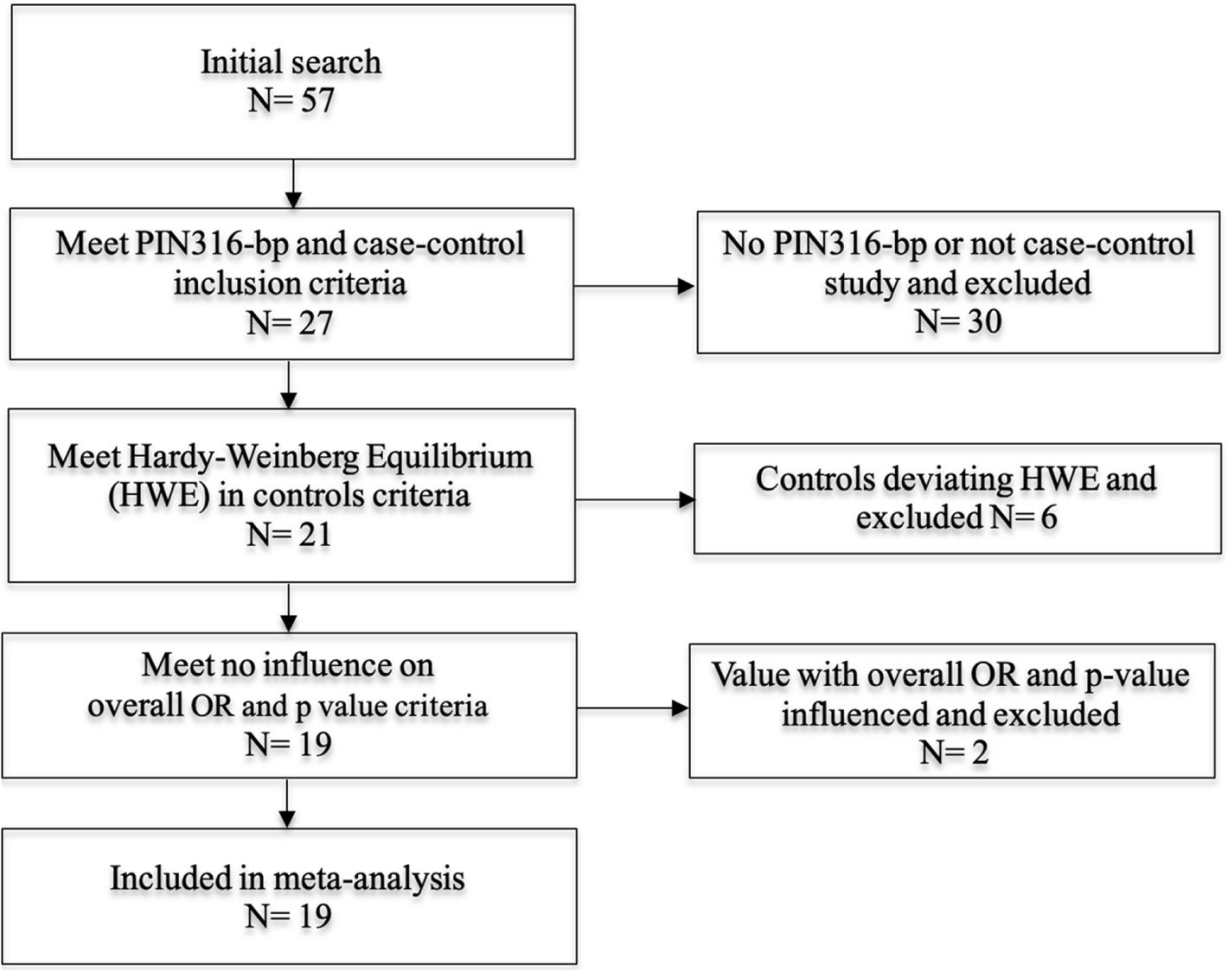
Flow chart of meta-analysis for exclusion/inclusion of studies

The criteria for selecting the articles were as follows: (1) Results reported about a case-control study, study published as an original study evaluating the association between PIN3 16-bp duplication polymorphism of *TP53* and the risk of breast cancer; (2) No deviation from Hardy-Weinberg Equilibrium (HWE) in controls; (3) No influence on the pooled odds ratio (OR) and *p*-values (Fig. 1); and (4) Full text available. Two investigators independently reviewed the abstracts of the initial search and assessed each article for inclusion in the meta-analysis.

#### Data ext***raction***

The following data were extracted from all eligible studies: first author’s name, year of publication, study population, sample size, genotypic and allelic distribution by two independent investigators (add the initials of the two extractors). These data were compared to find a consensus. A third investigator resolved any conflict.

### Statistical analysis

Review Manager Software was used to analyze the data. The Chi-squared test with the value of *P* < 0.05 was carried out to evaluate the Hardy-Weinberg equilibrium in the controls. The association of PIN3 16-bp duplication polymorphism with the risk of breast cancer in the dominant, recessive and additive models was measured by ORs with 95% CI. An inconsistency (I^2^) test was performed to detect heterogeneity [25]. If I^2^ <50% (absence of heterogeneity), the fixed effect model (FEM) was chosen as a pooling method; otherwise, if I^2^ >50% (presence of heterogeneity), the random effect model (REM) was maintained. The addition and/or deletion of any study that modifies the value of the pooled OR ± 1 was done to assess the sensitivity of the meta-analysis. The funnel curve was used to identify the publication bias.

## Results

### Case control study

We evaluated the association between PIN3 16-bp duplication polymorphism of *TP53* and the risk of breast cancer in Malian women. The demographic, clinical, and pathological characteristics of the patients are shown in Table 1. The mean age of cases and controls was 43.72 ± 3.14 and 43.90 ± 2.92 years, respectively. Most of the patients had cancer in the left breast. Multiparity was reported in 75.5% of cases, breastfeeding in 88.3%, no family history of breast cancer in 86.6%, no history of benign breast disease in 90.0%, absence of obesity in 68.3% and no history of smoking in 88.3% of the cases. Invasive ductal carcinoma forms were more prevalent than any others histological form of breast cancer (Table 1). Patients with PIN3 16-bp duplication (A2A2) of *TP53* were more likely to have an invasive ductal carcinoma form, T3 stage tumor size, node involvement (N0 and N1), and M0 metastasis status compared to patients with the A1A1 or A1A2 genotype. We found no correlation between the PIN3 16-bp duplication polymorphism and the clinical features of participants except histological type (*p* = 0.04).

**Table 1:** Distribution of the PIN3 16-bp duplication polymorphism of *TP5*3 according to the clinicopathological characteristics in Malian breast cancer

Clinical parameter	N (%)	PIN3 16-bp duplication			X^**2**^	*P* value
		A1A1%	A1A2%	A2A2%		
**Mean age at diagnosis**	43.72 ± 3.14				2.41*	0.12
≤ 40 years of age	29 (48.3)	11 (37.9)	12 (41.4)	6 (20.7)		
> 40 years of age	31 (51.7)	16 (51.6)	13 (41.4)	2 (6.5)		
**Localization**					1.98	0.74
Right breast	19 (31.7)	7 (36.8)	9 (47.4)	3 (15.8)		
Left breast	37 (61.7)	19 (51.4)	14 (37.8)	4 (10.8)		
Bilateral	4 (6.6)	1 (25.0)	2 (50.0)	1 (25.0)		
**Use of contraceptives**					0.56*	0.45
No	45 (75.0)	18 (40.0)	25 (55,6)	2 (4.4)		
Yes	15 (25.0)	9 (60.0)	–	6 (40.0)		
**Menopausal status**					3.15	0.53
Pre-menopausal	11 (18.3)	6 (54.5)	4 (36.4)	1 (9.1)		
Post-menopausal	20 (33.3)	10 (50.0)	9 (45.0)	1 (5.0)		
Fertile women	29 (48.3)	11 (37.9)	12 (41.4)	6 (20.7)		
**Parity**					7.33	0.12
Nulliparity	6 (10.0)	–	5 (83.3)	1 (16.7)		
Primiparity	9 (15.0)	3 (33.3)	4 (44.4)	2 (22.2)		
Multiparity	45 (75.5)	24 (53.3)	16 (35.6)	5 (11.1)		
**Breastfeeding**					0.50*	0.48
Yes	53 (88.3)	26 (49.1)	19 (35.8)	8 (15.1)		
No	7 (11.7)	1 (14.3)	6 (85.7)	–		
**Family history of BC**					0.64*	0.42
Yes	8 (13.3)	4 (50.0)	4 (50.0)	–		
No	52 (86.7)	23 (44.2)	21 (40.4)	8 (15.4)		
**Personal history of benign breast disease**					1.69*	0.19
Yes	6 (10.0)	4 (66.7)	2 (33.3)	–		
No	54 (90.0)	23 (42.6)	23 (42.6)	8 (14.8)		
**Obesity**					0.43	0.81
Yes	19 (31.7)	8 (42.1)	9 (47.4)	2 (10.5)		
No	41 (68.3)	19 (46.3)	16 (39.0)	6 (14.6)		
**Smoking**					0.20*	0.65
Passive smoking	7 (11.7)	3 (42.9)	4 (57.1)	–		
No	53 (88.3)	24 (45.3)	21 (39.6)	8 (15.1)		
**Histological type**					4.14*	0.04
Invasive ductal carcinoma	56 (93.3)	23 (41.1)	25 (44.6)	8 (14.3)		
Others	4 (6.7)	4 (100.0)	–	–		
**Tumor size**					5.63	0.46
T1	1 (1.7)	–	1 (100.0)	–		
T2	10 (16.7)	5 (50.0)	5 (50.0)	–		
T3	41 (68.3)	18 (43.9)	15 (36.6)	8 (19.5)		
T4	8 (13.3)	4 (50.0)	4 (50.0)	–		
**Nodal involvement**						
N0	36 (60.0)	16 (44.4)	16 (44.4)	4 (11.1)	6.05	0.41
N1	16 (26.7)	5 (31.3)	7 (43.8)	4 (25.0)		
N2	7 (11.7)	5 (71.4)	2 (28.6)	–		
N3	1 (1.7)	1 (100.0)	–	–		
**Metastasis**					0.91*	0.34
M0	55 (91.7)	24 (43.6)	23 (41.8)	8 (14.5)		
M1	5 (8.3)	3 (60.0)	2 (40.0)	–		

*X*^*2*^ Chi-squared test, *P p-*value, * Chi-squared test two-sided, *N* Number, *BC* Breast cancer, *A1A1* Wild-type, *A1A2* heterozygous, *A2A2* homozygous mutant, *%* Percentagwe, Other histological type: Glycogen-rich clear cell carcinoma, lobular carcinoma in situ, Moderately differentiated adenocarcinoma and infiltrating adenocarcinoma.

#### PIN3 16-bp duplication polymorphism of *TP53* and breast Cancer risk

Table 2 shows the distribution of PIN3 16-bp duplication polymorphism of the *TP53* in the cases according to the genetic models. The genotypic distribution PIN3 16-bp duplication polymorphism did not deviate from the Hardy-Weinberg equilibrium both in the cases (X^2^ = 0.33, *p* = 0.57) and in the controls (X^2^ = 2.76, *p* = 0.10). The heterozygous genotype (A1A2) was associated with an increased risk of breast cancer with (OR = 2.25, 95% CI = 1.01–5.01 and *p* = 0.04). When we extended the analysis to the different genetic models, we noted that the dominant model (A1A2 + A2A2 vs. A1A1: OR = 2.26, 95% CI = 1.08–4.73, *p* = 0.02) and the additive model (A2 vs A1: OR = 1.87, 95% CI = 1.05–3.33, *p* = 0.03) of PIN3 16-bp duplication polymorphism was significantly associated with the risk of breast cancer (Table 2).

**Table 2:** Association of genetic models of PIN3 16-bp duplication polymorphism of *TP53* with breast cancer risk

Genotype/Allele	Cases	Controls	OR (95% CI)	*P*
	*N* = 60	N = 60		
**A1A1**	27 (45.0)	39 (65.0)	Reference	
**A1A2**	25 (41.7)	16 (26.7)	2.25 (1.01–5.01)	0.04
**A2A2**	8 (13.3)	5 (8.3)	2.31 (0.68–7.83)	0.17
**A2A2 + A1A2**	33 (55.0)	21 (35.0)	2.26 (1.08–4.73)	0.02
**A1A1 + A1A2**	52 (86.7)	55 (91.7)	Reference	
**A2A2**	8 (13.3)	5 (8.3)	1.69 (0.52–5.50)	0.38
**A1**	79 (65.8)	94 (78.3)	Reference	
**A2**	41 (34.2)	26 (21.7)	1.87 (1.05–3.33)	0.03

*N* Number, *CI* confidence Interval, *P p*-value, A2A2 + A1A2 vs. A1A1: Dominant model, A2A2 vs. A1A1 + A1A2: Recessive model; A2 vs. A1: Additive model.

### Meta-analysis study

#### Characteristics of included studies

A total of 19 articles reporting case-control studies that investigated PIN3 16-bp duplication polymorphism and breast cancer risk and meeting the inclusion criteria were selected to perform the meta-analysis (Table 3, Additional file 1). Thirty studies that have not addressed PIN3 16-bp duplication of *TP53,* 6 studies deviating from HWE, as well as 2 studies [38, 39] which influenced the OR and *p* values pooled were excluded (Fig. 1).

**Table 3:** Summary of studies included in meta-analysis

Reference	Population	Cases				Controls				
		N	A1A1	A1A2	A2A2	N	A1A1	A1A2	A2A2	HWE
Present study	Mali	60	27	25	8	60	39	16	5	0.10
Akkiprik et al. 2009 [18]	Turkey	97	59	35	3	107	61	43	3	0.15
Buyru et al. 2007 [26]	Turkey	115	83	28	4	63	47	15	1	0.87
Cherdyntseva et al. 2012 [27]	Russia	296	227	68	1	196	145	50	1	0.13
Costa et al. 2008 [17]	Portugal	191	122	56	13	216	147	65	4	0.29
De Vecchi et al. 2008 [28]	Italy	350	233	103	14	352	256	87	9	0.62
Gaudet et al. 2007 [29]	USA (M)	578	404	157	17	390	272	108	10	0.85
Gohari-Lasaki et al. 2015 [23]	Iran	100	53	38	9	100	60	37	3	0.34
Guleria et al. 2012 [30]	India	80	43	30	7	80	53	25	2	0.64
Hao et al. 2018 [31]	Chine	254	230	24	0	252	227	25	0	0.41
Hrstka et al. 2009 [32]	Island	117	81	32	4	108	81	24	3	0.46
Morten et al. 2019 [20]	Australia	1304	986	289	29	436	325	104	7	0.67
Pouladi et al. 2014 [33]	Iran	221	135	69	17	170	107	51	12	0.10
Sharma et al. 2014 [7]	India	200	134	52	14	200	137	55	8	0.41
Suspitsin et al. 2003 [34]	Russia	529	408	108	13	249	187	56	6	0.47
Trifa et al. 2010 [35]	Tunisia	159	98	56	5	132	86	41	5	0.97
Vymetalkova et al. 2015 [36]	Czech	705	474	164	24	611	421	172	18	0.93
Wang-Gohrke et al. 2002 [16]	Germany	563	370	173	20	549	391	145	13	0.92
Weston et al. 1997 [37]	USA (M)	99	60	36	3	185	127	54	4	0.52

*M* Mixed, *N* Number

#### Quantitative analysis

**Fig. 2:**
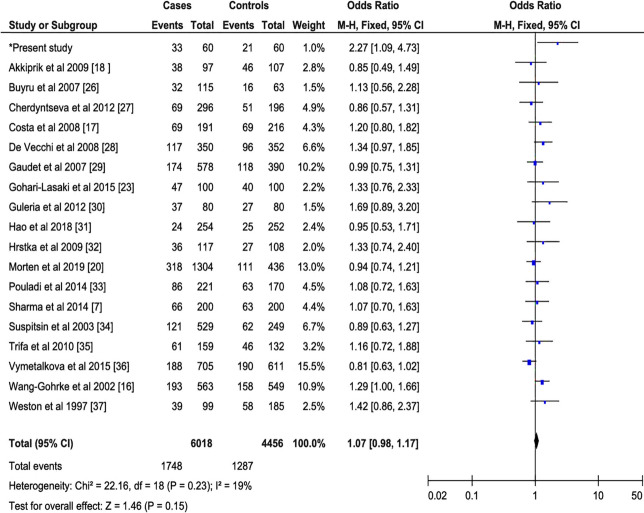
Forest plots of the relationship between PIN3 16-bp duplication polymorphism of the *TP53* and breast cancer in the dominant model. The black diamond denotes the pooled OR; black squares indicate the OR in each study with square sizes inversely proportional to the standard error of the OR; and horizontal lines represent the 95% CI

**Fig. 3:**
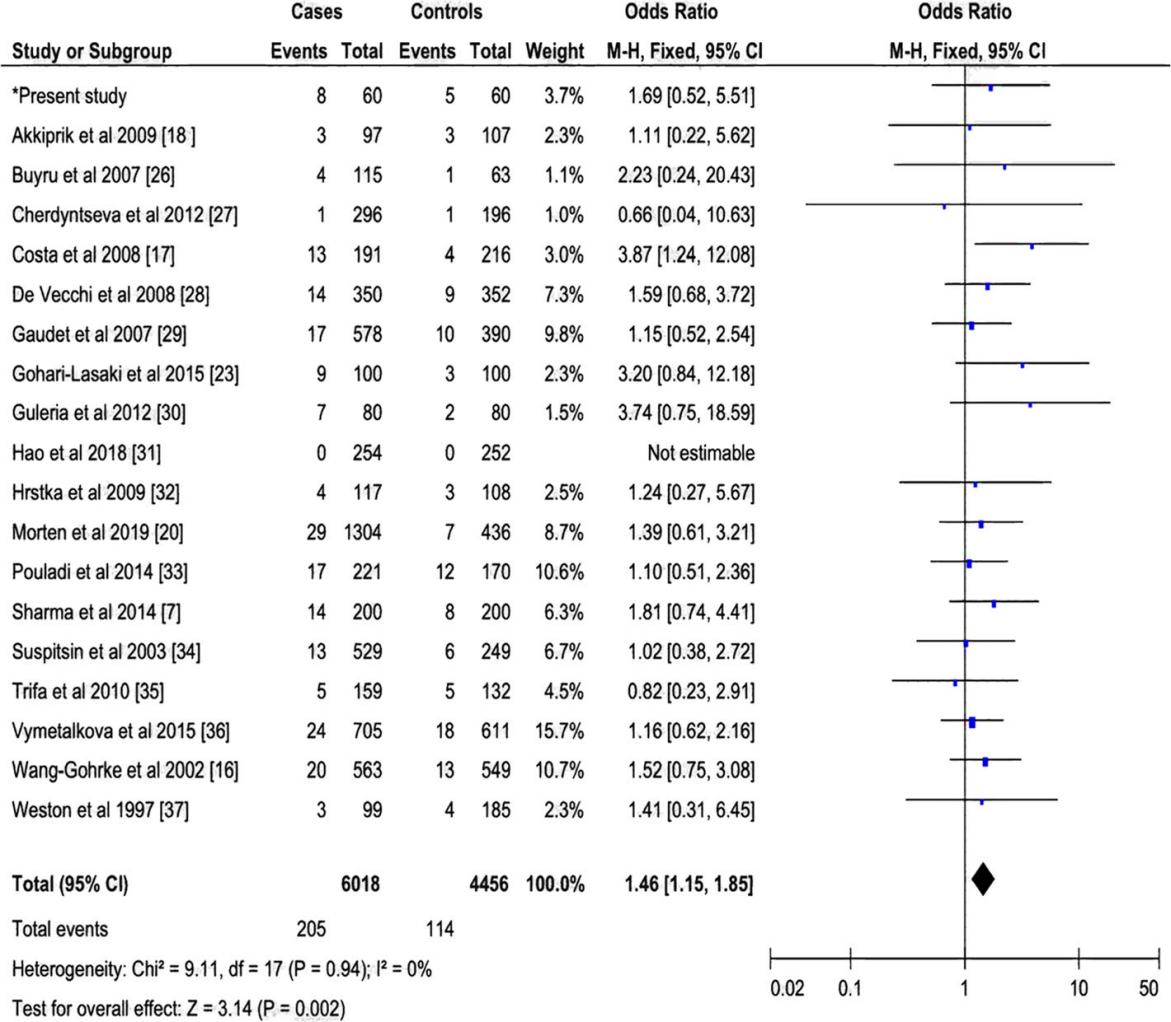
Forest plots of the relationship between PIN3 16-bp duplication polymorphism of the *TP53* and breast cancer in the recessive model. The black diamond denotes the pooled OR; black squares indicate the OR in each study with square sizes inversely proportional to the standard error of the OR; and horizontal lines represent the 95% CI

**Fig. 4:**
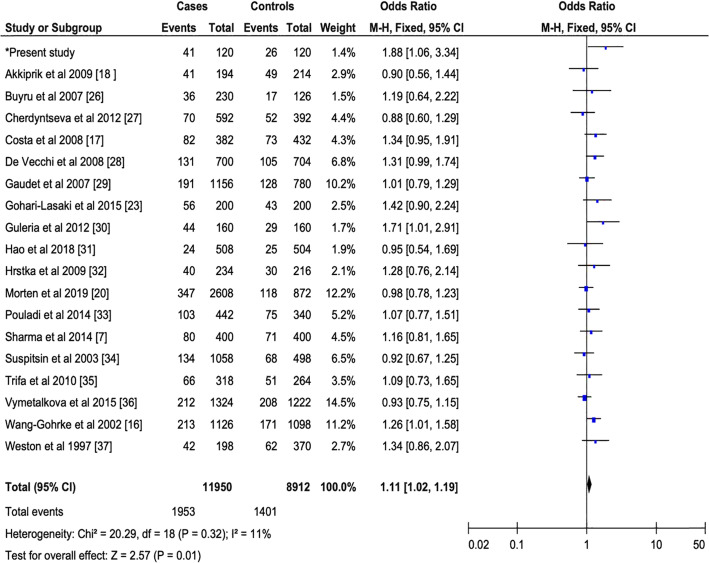
Forest plots of the relationship between PIN3 16-bp duplication polymorphism of the *TP53* and breast cancer in the additive model. The black diamond denotes the pooled OR; black squares indicate the OR in each study with square sizes inversely proportional to the standard error of the OR; and horizontal lines represent the 95% CI

This meta-analysis showed a significant association between PIN3 16-bp duplication polymorphism and breast cancer risk in recessive (Fixed effect model (FEM): OR = 1.46, 95% CI = 1.15–1.85; *p* = 0.002) and additive (FEM: OR = 1.11, 95% CI = 1.02–1.19; *p* = 0.01) models, but not in the dominant model (FEM: OR = 1.07, 95% CI = 0.98–1.17; *p* = 0.15). Figures 2, 3, and 4, show the forest plots of OR for breast cancer in the dominant, recessive and additive models of PIN3 16-bp duplication polymorphism of the *TP53*, respectively. Figure 2**.**

#### Sensitivity analysis

The stability of the results was assessed by a sensitivity analysis. We have noted a significant association between the PIN3 16-bp duplication polymorphism and the risk of breast cancer in the recessive (Fig. 3) and additive (Fig. 4) models, except the dominant model (Fig. 2), Furthermore, the one by one elimination of eligible studies did not influence the values of the pooled OR effect in the different genetic models.

#### Sources of heterogeneity

After the non-inclusion of articles with HWE-deviation in controls, we noted a lack of heterogeneity in the dominant (**I**^**2**^ = 19%, *P* = 0.23), recessive (**I**^**2**^ = 0%, *P* = 0.94) and additive (**I**^**2**^ = 11%, *P* = 0.32) models between PIN3 16-bp duplication polymorphism and breast cancer risk (Figs. 2, 3, and 4).

#### Publication Bias

**Fig. 5:**
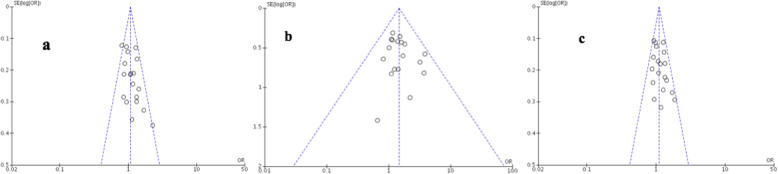
Funnel plots of **a** dominant, **b** recessive and **c** additive models precision by OR

A funnel plot was used to assess publication bias. After the elimination of studies that did not meet the inclusion criteria followed by the sensitivity analysis, no publication bias was observed in the recessive and additive models. However, a slight asymmetry was detected in the dominant model (Fig. 5).

## Discussion

n the present study, we noted a positive correlation of the PIN3 16-bp duplication polymorphism of *TP53* with the histological type of breast cancer. Similar results have been found in the Iranian population by Faghani et al. who reported a correlation between invasive ductal breast cancer and the PIN3 duplication polymorphism at 16 bp [40]. Contrary to our observations, studies carried out in the Moroccan, Croatian and Czech populations have not found any link between histological types and mutations in this gene [19, 32, 38]. These contradictory results may be explained by the ethnic and geographic origin.

Our results show that the PIN3 16-bp duplication polymorphism is significantly linked to the breast cancer risk in the Malian population.

We found that heterozygous, dominant and A1A2 additive models were significantly associated with an increased risk of breast cancer. However, the results of various studies regarding the association between the PIN3 16-bp duplication of *TP53* and the risk of breast cancer are contradictory. Similar to our results, Faghani et al. and Wu et al. reported that the A1A2 genotype is associated with the risk of breast cancer [40, 41] On the other hand, others studies have found no association between this genotype and the risk of breast cancer [18, 38, 39]. However, we noted that the A2A2 genotype was not associated with the development of breast cancer in our population. This observation was similar to those previously reported by in Morocco [19], in Iran [40], and Poland [42] but contradictory with the result obtained in Portugal [17]. In addition, we noted that the A2 allele was associated with the risk of breast cancer, which was consistent with the results of many authors [30, 40] but different from the results reported by others [31, 36]. The differences between studies may be explained by several factors such as sample size, race, ethnic differences, genetic background, environmental factors and heterogeity between the studies.

The meta-analysis, which included 6018 breast cancer patients and 4456 controls revealed an increased-risk of breast cancer with the recessive and additive models of PIN3 16-bp duplication. Two previous meta-analyzes, one covering 19 studies with 4479 cases and 4683 controls [41] and the other covering 9 studies with 2715 cases and 2595 controls [21] showed that the recessive model was associated with the risk of breast cancer. However, another meta-analysis of 6 studies with 2018 cases and 1748 controls revealed an inverse association [22], but the number of studies included and the sample size for this study were relatively small. Compared to our results, all these meta-analyzes found a significant genetic association between the additive model and breast cancer [21, 22, 41]. The mechanism associating A2 with breast cancer is not yet fully established, certain factors have been discussed. There is some evidence linking A2 status of differential expression of different p53 isoforms in lymphoblastoid cell lines, thereby causing alteration in mRNA [13, 43, 44]. Indeed, the influence of A2 allele on the alternative splicing of p53 protein causes an instability of the transcripts or proteins with modified functions. Many investigators have reported the existence of linkage disequilibrium between 6-bp duplication and other variants of *TP53* such as codon 72 or p.Arg72Pro, intron 6 [31, 45]. The codon 72 Arg/Pro, intron 3 16-bp duplication and intron 6 G > A *TP53* haplotype was associated with the ability to repair DNA in lymphoblastic cell lines and apoptic reduction [21, 46]. Thus, the polymorphisms of *TP53* could affect the activity of *p53* by triggering the process of carcinogenesis.

This study has some limitations such as small sample size, lack of hormonal receptors tests and subgroup analyzes in the meta-analysis. Another limitation is the collection of data limited to the demographic parameters and history of the disease in controls.

## Conclusions

The present study made it possible to establish for the first time the distribution of alleles and genotypes of PIN3 16-bp duplication polymorphism of *TP53* in the Malian population and to understand the relationship between this gene and the risk of breast cancer. Our results have shown that this polymorphism is not only associated with the histological type, but also is with the risk of breast cancer in Malian population. In addition, the meta-analysis carried out confirmed our findings.

## Supplementary information

**Additional file 1.** Availability of all data and references with PubMed accession numbers

## Data Availability

The datasets generated and/or analyzed in the Malian population study are available from the corresponding author upon reasonable request and with the permission of FMPOS Ethics Committee. The meta-analysis dataset analyzed is available in the additional file 1.

## Abbreviations

AS-PCR: Allele Specific PCR
CHU: University Hospital Center
CI: Confidence Interval
FEM: Fixed effect model
HWE: Hardy-Weinberg Equilibrium
LMICs: Low- and middle-income countries
OR: Odd ratio
REM: Random effect model
USTTB: University of Science, Technique, and Technologies at Bamako
