# Supplementary material for: Association of PIN3 16-bp duplication polymorphism of TP53 with breast cancer risk in Mali and a meta-analysis
Source: BMC Med Genet. 2020 Jul 3;21:142. doi: 10.1186/s12881-020-01072-4 (PMC7333399; doi:10.1186/s12881-020-01072-4)
Supplement: Supplementary file 1 — Additional file 1. Availability of all data and references with PubMed accession numbers [file 12881_2020_1072_MOESM1_ESM.docx]

**Additional file 1.** Availability of all data and references with PubMed accession numbers

| **Authors** | **Accession (PubMed)** |
| --- | --- |
| Present study | - |
| Akkiprik et al 2009 [18] | PMID: 19048399 |
| Buyru et al 2007 [26] | PMID: 17574348 |
| Cherdyntseva et al 2012 [27] | PMID: 21838531 |
| Costa et al 2008 [17] | PMID: 18230179 |
| De Vecchi et al 2008 [28] | PMID: 18640791 |
| Gaudet et al 2007 [29] | PMID: 17624591 |
| Gohari-Lasaki et al 2015 [23] | PMID: 25854391 |
| Guleria et al 2012 [30] | PMID: 22994752 |
| Hao et al 2018 [31] | PMID: 30309383 |
| Hrstka et al 2009 [32] | PMID: 19787241 |
| Morten et al 2019 [20] | PMID: 30430302 |
| Pouladi et al 2014 [33] | PMID: 24761875 |
| Sharma et al 2014 [7] | PMID: 25169539 |
| Suspitsin et al 2003 [34] | PMID: 12471629 |
| Trifa et al 2010 [35] | PMID: 20233677 |
| Vymetalkova et al 2015 [36] | PMID: 2622648 |
| Wang-Gohrke et al 2002 [16] | PMID: 11927843 |
| Weston et al 1997 [37] | PMID: 9037561 |
